# Germination rate is the significant characteristic determining coconut palm diversity

**DOI:** 10.1093/aobpla/pls045

**Published:** 2012-12-28

**Authors:** Hugh C. Harries

**Affiliations:** Coconut Time Line, Weymouth, Dorset DT3 5NP, UK

## Abstract

**Rationale:**

This review comes at a time when *in vitro* embryo culture techniques are being adopted for the safe exchange and cryo-conservation of coconut germplasm. In due course, laboratory procedures may replace the options that exist among standard commercial nursery germination techniques. These, in their turn, have supplanted traditional methods that are now forgotten or misunderstood. Knowledge of all germination options should help to ensure the safe regeneration of conserved material.

**Scope:**

This review outlines the many options for commercial propagation, recognizes the full significance of one particular traditional method and suggests that the diversity of modern cultivated coconut varieties has arisen because natural selection and domestic selection were associated with different rates of germination and other morphologically recognizable phenotypic characteristics. The review takes into account both the recalcitrant and the viviparous nature of the coconut. The ripe fruits that fall but do not germinate immediately and lose viability if dried for storage are contrasted with the bunches of fruit retained in the crown of the palm that may, in certain circumstances, germinate to produce seedlings high above ground level.

**Significance:**

Slow-germinating and quick-germinating coconuts have different patterns of distribution. The former predominate on tropical islands and coastlines that could be reached by floating when natural dispersal originally spread coconuts widely—but only where tides and currents were favourable—and then only to sea-level locations. Human settlers disseminated the domestic types even more widely—to otherwise inaccessible coastal sites not reached by floating—and particularly to inland and upland locations on large islands and continental land masses. This review suggests four regions where diversity has been determined by germination rates. Although recent DNA studies support these distinctions, further analyses of genetic markers related to fruit abscission and germination are recommended.

## Abstract (precis)

Using information and DNA analysis data accessable on the internet, this review builds on a germination and taxonomy study of the coconut palm published in Annals of Botany in 1981, resolves earlier issues, and opens up new lines of investigation.

## Introduction

As might be expected for a plant like the coconut palm (*Cocos nucifera* L.), that is so important across cultures and countries but which cannot be cloned or vegetatively propagated, there are many ways to produce seedlings for planting (Menon and Pandalai 1958; Child 1974; Ohler 1999). This has made germination a focus for the earliest scientific research (Winton 1901; Kirkwood and Gies 1902) to the present day (Konan *et al.* 2011; D'Amato *et al.* 2012). In addition to its necessary role in propagation, the fruit is also the source of the coconut's main economic products. To mention a few only, when immature it provides tender-nut water, currently a popular athletic and health drink, when mature the husk is a source of ecofriendly geotextiles and cocopeat, the shell can be converted to a superior quality activated carbon, the fresh kernel can be desiccated for confectionery or variously processed to extract coconut oil which has a multitude of edible and industrial uses—from ‘milk’ or ‘cream’ in cookery, to candles, soaps and shampoos, high explosives, biofuels and nutriceuticals. This list is not exhaustive but the most important role, and the one that concerns this account, is the part that the coconut fruit has played in determining the diversity of this pan-tropical crop plant.

## Coconut germination

### The viviparous and recalcitrant seednut

The coconut seed, or seednut, is the entire fruit, and botanically it is a drupe, consisting of pericarp and mesocarp (husk), endocarp (shell) and testa enveloping the mature endosperm (kernel) which, importantly, has a central cavity (air space) containing residual liquid endosperm (water). The large size and heavy weight of the seednut prevent dissemination by animals or birds but the fibrous husk and the air space give it the necessary buoyancy for dispersal by floating. According to Corner (1966; cited by Child 1974), the peg-like embryo, embedded in the kernel beneath the germ pore in the shell (the ‘soft eye’), never really stops growing. This effectively makes the coconut viviparous (live-bearing) and, as it is no longer viable if it is dried for storage (as copra), it is therefore classified as recalcitrant (Chin and Roberts 1980).

The fruit takes ∼11–14 months to develop after the receptive female flower is successfully pollinated and before the growing point of the embryo penetrates the germ pore (Marar and Varma 1958). At that moment, the embryo also begins to enlarge internally, to eventually fill the cavity completely with a sponge-like haustorium (Sugimura 1998). This produces the enzymes that convert the oil in the kernel into nutrients that are then absorbed to support growth (Balasubramaniam *et al.* 1973; Manjula *et al.* 1993, 1995*a*, *b*; Balachandran and Arumughan 1995) until leaves and roots emerge through the husk and expand in daylight for photosynthesis to begin.

Since the husk inconveniently conceals the earliest stage of germination through the germ pore, the emergence from the husk of the tip of the young shoot (often described as a ‘crow's beak’) is usually recognized as the date of germination for record-keeping purposes, although the date of harvest or collection, the duration of any storage or treatment period and the date of setting in the nursery are also important when different germination methods need to be compared (Harries 1981, 1983). Depending on the variety and climate, the shoot may not emerge until some time after the mature coconut has dropped from the palm or been harvested. Within the husk, the growing point of the embryo develops a plumule and root initials, which eventually emerge through the husk, the time taken depending on the husk thickness.

Coconut seednuts germinate easily in warm, humid conditions and will sprout and grow naturally wherever they fall—with the unfortunate consequence that neglected coconut groves can become badly overcrowded. Even the most efficiently maintained groves need to be replanted before they become over-aged and economically unproductive. Germination methods have been developed to produce a flush of uniformly vigorous seedlings in large quantities. These must establish well, grow robustly, flower precociously, fruit heavily and regularly for many years and, in every respect, be an improvement over the previous generation that they replace (Pandittesekere 1914; Pieris 1937; Menon and Pandalai 1958; Harries 1983; Reddy *et al.* 2001), and numerous ways to accelerate and maximize total germination have been suggested (see options below).

In the absence of any means of cloning high-yielding coconut palms (tissue culture is currently restricted to zygotic embryos), research has developed *in vitro* techniques by which embryos can be germinated. This began with certain types, known in the Philippines as *makapuno* (and under other names in other Asian countries) in which the unusual, jelly-like, endosperm does not support normal germination. Now, to overcome the viviparous and recalcitrant nature of the seednut, propagation of excised embryos is becoming important for the safe international transfer and cryo-conservation of all coconut germplasm. This will probably involve the same options for the initial seednut selection and the eventual growing-on in polybag nurseries (described in options below) but with the intermediate options being replaced by appropriate *in vitro* steps for laboratory culture (N'Nan *et al*. 2012).

### Germination options

Seednuts may be allowed to drop when ready, before being collected, or are harvested by being cut from the palm at a specific stage of development (11th or 12th ‘month’ or when the skin of the husk begins to change colour). They may be stored before setting, or set without undue delay. Sometimes a thin slice of husk is removed or a cavity is made in the husk to receive micro-nutrients or the husk may be partially or entirely removed for other purposes. Seednuts may be soaked in water or in nutrient solution, sprinkler-irrigated or only set at the start of, or during, a rainy season. Depending on their shape they may be set on their broadest side or on an edge, or on their base with the eye-end uppermost. Set to half-depth in prepared nursery beds, they may be close together or given space, with or without shade, mulch or irrigation, and left for 7–9 months before being lifted as bare root seedlings for field planting. Alternatively, they may be laid loosely on the ground in a pre-nursery (preferably irrigated) and transferred individually, when a sprout appears, to nursery beds (as above) or into polybags, with the option to be field planted when much bigger or older than bare root seedlings.

### Commercial propagation techniques

#### Nurseries

Coconuts are almost always germinated in nurseries. Planting seednuts directly in the field is not recommended, since not each one germinates. In the nursery, the young seedlings receive protection from browsing and grazing animals, freedom from weed competition and from being excessively over-shaded by older palms or by intercrops. It is usually considered a sign of poor management if fallen nuts are allowed to sprout beneath an existing canopy, so ripe nuts need to be regularly collected, if they have fallen to the ground, or regularly harvested from the palm if they do not fall. This also reduces the risk of praedial larceny (theft of agricultural produce).

#### Harvesting

Immature tender-nuts, for drinking, must be carefully lowered to the ground, to avoid splitting, but ripe seednuts fall naturally with little or no damage, or can be harvested from ground level by cutting off individual fruit or entire bunches, using a knife or a hook on a bamboo or an aluminium pole. Skilled harvesters (sometimes employing trained monkeys) can visually recognize different stages of fruit development. Often referred to in India and Sri Lanka as ‘11-month’ or ‘12-month’ nuts, and implying the time elapsed since flowering, the actual signs that indicate readiness are shrinkage of the husk and a progressive change in skin colour (at 11 months) and (at 12 months) a dry brown skin but a calyx that retains its fresh colour (shades of green, bronze, red or yellow).

The one specific germination criterion for both 11- and 12-month categories is the splashing that can be heard (and felt) when the nut is shaken because some of the water in the nut cavity has been absorbed, leaving an air space. Fresh coloured and heavy fruit that do not splash are rejected as immature. Completely dry brown fruit that do not splash are also rejected as they may already have a sprout beneath the husk and a haustorium in the nut (Aiyadurai 1956). If used as seednuts they may produce distorted and late germinating seedlings by being set upside down in the nursery (Harries 1981).

#### Storage

In some circumstances, for instance if seednuts have to be harvested during a dry season, they may be stored under shelter for some weeks, and possibly up to 4 months according to John and Narayana (1942), as cited by Menon and Pandalai (1958). This is possible with slow-germinating populations found in India and Sri Lanka where it would allow 11 month nuts to ripen but, for obvious reasons, early germinating varieties should be set without undue delay (Satyabalan 1985; Remison and Mgbeze 1988).

#### Preparation

It has become the practice in some communities, particularly those with thick husked, slow-germinating varieties, to remove a thin slice of husk before setting in the nursery (Ugbah and Akpan 2003). This is thought to allow moisture to penetrate and may have originated from experiments where a cavity was made in the husk to receive micro-nutrients (such as manganese and iron) which are unavailable on alkaline soils (Pomier 1967; de Silva and Aputharajah 1977; Sumathykutty Amma 1964; Thomas 1974). There is a risk of wounding the developing sprout if cut too deeply in thin husked, early germinating varieties, weakening the support at the base of the stem and breaking the connection to the supply of nutrients from the haustorium, or allowing the entry of disease spores.

The additional labour cost of trimming must also be taken into consideration and may only be acceptable for special purposes. For example, partial husk removal was adopted to reduce weight and cost for air transport (Whitehead 1966, 1968), and for experiments to improve root establishment (Kenman 1973; Foale 1993); however, total husk removal is a necessary preliminary step in extracting embryos for micro-propagation by tissue culture and for cryopreservation (de Guzman and del Rosario 1964; Kartha 1981).

Soaking over-mature, dry brown (post-12 months) seednuts with water or with nutrient solution does not revive those where the kernel is already drying to copra (Zuniga *et al.* 1971; Aiyadurai 1956). Soaking seednuts by floating (Marar and Shambhu 1961) in a tank, pond or, possibly, a lagoon, prior to setting in the nursery is less convenient than sprinkler-irrigation after setting. Otherwise, setting in the nursery is best done at the start of, or during, a rainy season (and hence a reason for storage).

#### Nursery requirements

Where possible, nurseries should be established in, or close to, the field that is to be planted, to minimize handling and transport of the seedlings. Preparation of the nursery will depend on the type of seedling to be produced (bare root or polybag) and it is also necessary to make allowance for the shape of the seednut (angular or spherical). In all situations a well-drained sand or loam soil is preferred, with moisture-retentive materials, such as cocopeat (coir pith or coir dust) added, perennial weeds removed and termites, etc., controlled.

For bare root seedlings, nursery bed soil needs to be loose so that seednuts laid horizontally, at no more than half their depth, can be tilted slightly forward towards the eye end so that the water in the nut moistens the embryo and the thickest part of the husk gives support to the emerging sprout. Long, angular seednuts should be set on their broadest face (the natural position after falling or floating). Setting seednuts on an angled edge is sometimes recommended but entails additional work to open shallow trenches or make ridges. A mulch can be applied to just cover the seednuts allowing rain or irrigation to settle it so that the top of the nut is exposed, but fertilizer is not necessary. Temporary light shade can be given in very hot, dry weather.

#### Transplanting

If nuts are set close together (not quite touching) they must be transplanted to their final field positions before the seedlings compete for light and air (at ∼30 weeks, with three leaves and four or five emerging roots; Child 1974); if set with more space they still need to be transplanted (at ∼9 months) before rooting becomes too extensive. A narrow-bladed, sharp-edged spade (or a machete) will cut the roots cleanly and a number of seedlings can be carried in each hand (taking care not to dislocate the connection with the haustorium by rough handling) and they should be planted with the minimum of delay (or kept in cool, moist shade if delay is unavoidable). Bare root seedlings suffer if they become overgrown in the nursery on occasions when field planting is delayed but seedlings in polybags can be fertilized, irrigated and moved further apart to give extra space and extra time in the nursery.

Seednuts, which are very spherical, can be set on their base with the eye-end uppermost and, possibly, directly into half-filled polybags, adding more soil when the sprout appears. Some bags may be wasted because total germination is never 100 % but germination is quicker and, after field planting, polybag plants come into bearing more quickly than bare root seedlings.

#### Pre-nurseries

An option that avoids storage and overcrowded seed beds is to lay the harvested seednuts loosely on the ground in a sprinkler-irrigated (and possibly lightly-shaded) pre-nursery and as soon as crow's beak sprouts appear, and before roots emerge, transfer batches of seedlings to spaced nursery beds or individual polybags. This gives the chance to sort seedlings by age, size or colour, and can be especially useful to identify and eliminate off-types among hybrids. Any seednuts in nurseries or pre-nurseries that do not sprout after a specific time (three to four months, depending on variety) should be discarded, along with any chlorotic, contorted, damaged or otherwise unsuitable seedlings. The occasional poly-embryonic seedlings (twins, etc.) might have ornamental value and possible haploids should be offered to tissue culture laboratories.

### Traditional propagation techniques

At a time when coconut was reaching its apogee as the most important source of vegetable oil in international trade, five alternative germination options listed in a monograph (Menon and Pandalai 1958) were considered odd to the point of being defective. The seednuts were either (i) placed on the roofs of thatched houses during the monsoon and directly transplanted in the field when they sprout; (ii) heaped in shade and allowed to sprout; (iii) tied in pairs with a strip of husk split from each and then suspended from branches of trees or bamboo posts; (iv) tied round an upright pole in the open; or (v) simply allowed to remain where they fall. Yet, while these unusual options are unsuitable for modern commercial nurseries (Marar and Jayarajan 1963), the first four are traditional techniques that take advantage of a common factor—early germination—while the fifth is simply the natural dissemination process.

It is the purpose of this article to show that, far from being defective, the four ‘aerial’ options take advantage of the viviparous nature of the coconut and account for the different pattern of distribution of the quick-germinating domestic coconut from that of the slow-germinating naturally disseminated wild type.

#### Aerial germination

The option to germinate seednuts out of contact with the soil, by hanging them from a pole to sprout, was considered odd (Child 1974), defective (Menon and Pandalai (1958) and unsatisfactory (Marar and Jayarajan 1963). However, it was previously recommended as a good method in the Philippines that gave better control over the germination process (Copeland 1914, p. 118). Described in more detail at that time and illustrated, together with a similar method with nuts stacked round a pole, it was in use in more recent times in the Micronesian island of Yap (Anonymous 1960) and also illustrated and described. The necessary steps begin with the selection of the seednut, which is considered to be highly important. The selected seednuts are then strung by coconut husks to bamboo poles supported on bamboo frames at a proper height from ground and allowed to germinate. The germinated seednuts are prepared for planting at the sixth-leaf stage when the seedling is taken from the pole and the seednut is cut in half vertically in one sharp stroke with the stem and leaves remaining on the portion to be planted. Before planting, the haustorium (‘coconut apple’) and the residual endosperm are removed and the empty cavity is filled with soil of the local area where seed will be planted. Planting requires the prepared seedling to be placed securely at proper angle in a pre-dug planting hole where the stem stands erect after planting and the soil settles in hole by gradual action of wind and rain.

The method is claimed to be unique, because ground nurseries are not used, and the author (possibly Gabriel Gilrow, District Agricultural Extension Agent or Manny Sproat, the Territory's Director of Agriculture and Fisheries at that time) gives this explanation:

The seednuts are hung along a horizontal bar high above the ground for sprouting, to discourage development of roots prior to planting. It is believed that exposure to the sun in this manner develops vigor, and also, the off-ground sprouting system tends to reduce infestation by insects, or infection by disease. It is considered that transplanting the seed after the roots have developed in the ground would cause unnecessary setback in growth—that some injury, even though slight, is bound to result as the roots are removed from the earth. In the Yapese theory of coconut propagation, the development of the seedling is highly important. The properties of the nut itself are believed to be the key to superior coconut production. The traditional stage for selecting the seedling (from the bamboo-strung rows of nuts) is when the sixth leaf is fully developed. Broad leaves with short petioles are sought. Seedlings also are selected on the basis of straight, thick stems, and freedom from insect infestation or disease. Roots should be developed so that they are clearly visible, extending perhaps one-fourth inch out from the surface of the husk—and the number of roots should be the same as the number of leaves on the stem. The Yap method of cutting the nut in preparation for planting is probably the most unusual of all the various steps followed. The nut of the selected seedling is cut in half vertically—or diagonally—and the haustorium and endosperm removed from that portion of the nut to which the sprout or seedling is attached.

The removal of haustorium and residual endosperm does seem to distinguish the Yap method from any other and is at odds with current opinion that, after field planting, the kernel remaining inside the nut helps the young plants to survive periods of drought (Foale 1968). Nevertheless, the author of the Micronesian Reporter article asked ‘Who can say that this is not science?’ and concluded:

The various processes followed by the coconut growers of Yap have developed after experimentation, study and evaluation over the years—not with charts and papers, but with eyes and ears. The accumulated knowledge has been organized, and a system established. The results speak for themselves. Yap's coconuts bow second to none (Anonymous 1960).

The various modern, commercial, nursery methods and the Yap method of traditional, aerial, propagation differ widely but they share a common cause—which is the rate of germination. The obvious explanation is that slow germinators have thicker husks than fast germinators, and scientific analysis may yet reveal other physiological or biochemical factors, but thick husk has already been attributed to natural selection for dispersal by floating and thin husk to domestic selection and dissemination of coconuts with increased water content (Harries 1978). The contrasting natural and domestic environments are the circumstances where different rates of germination developed. The consequences of these processes have been to greatly influence the diversity of the cultivated coconut.

## Coconut diversity

### The prehistoric natural habitat

The coconut that evolved without human intervention could only become naturally established just above the tide line of beaches on newly emerged volcanic islands and atolls or favourable continental coastlines to which it could float. Without human intervention it could not reach or survive in inland or upland regions of large islands or continental land masses. In those situations human cultivators must clear and control competing vegetation, carry and plant seednuts or seedlings and prevent over-shading from more vigorous tropical rainforest trees.

With no cold winters on the warm, humid and sunny tropical beaches, which are the natural habitats for *C. nucifera*, dormancy is no advantage because individual coconut palms are capable of producing viable seednuts every month of every year during their perennial lifetime—the best part of a century—subject only to adequate groundwater or rainfall.

The first priority is for the mature fruit to drop from the palm directly to the ground and germinate to ensure a continuous presence of coconuts in the same location. Speed of germination is immaterial in this instance, since seedling development is restricted in the presence of the perennial parent palms. Fruits that may fall into a lagoon and float help to maintain homogeneity between populations on opposite sides of an atoll beyond the range of cross-pollination. More significantly, those fruits that drop into the ocean can float to establish similar coconut populations in new coastal locations (Harries 1981). To the floating coconuts in the second and third instances, quick germination would be a distinct disadvantage because, despite the palm's tolerance of salinity, seedlings will not survive if immersed in sea water (in contrast to mangrove, *Rhizophora* spp., or the Nipa palm, *Nypa fruticans*).

Coconuts float because of the fibrous husk and the air space in the nut cavity but any successful trans-lagoon or trans-oceanic dissemination requires a slow rate of germination. The thickness of the husk contributes both to floating ability and to slow germination. It has also been suggested that salt water absorbed in the husk may increase the matric (osmotic) potential and induce a degree of dormancy (Harries 1981) but this has not been experimentally validated.

Presumably starting from some smaller fruited primordial and ancestral form, the coconut evolved naturally into the large fruited coconut that can still be found today (Harries 1978). Fossils with *Cocos*-like features that have been found as far apart as Australia and New Zealand, India and South America confirm the ability to float but make it difficult to determine a precise origin. This wild type is considered to have become naturally established in favourable locations on islands fringing the east coast of Africa, south and southeast Asia to those of the western Pacific, before any of those places had been reached or settled in by humans.

#### Germination rate

The thick husked coconut with long, angular fruit having a high proportion of husk at the ends and in ridges along the length corresponding to the fundamental tricarpellate ovary has an egg-shaped (ovoid) nut inside with a thick shell, a kernel rich in oil and a small cavity that aids buoyancy. It never germinates viviparously on the palm but falls spontaneously when mature and takes from 60 to 220 days to achieve 90 % germination success (Whitehead 1965). Once the tide delivers it on a beach the angular shape prevents the fruit from rolling and shifting in the surf so that it remains there to root and does not easily wash away again.

#### Locations

Before any human involvement these coconuts could only be found on beaches, where tides and currents carried them. Atolls in the Indian and the western Pacific oceans were ideal and represent the first centre of diversity but the naturally disseminated coconut could not reach inland or upland areas of high, uplifted or volcanic islands. Small groves that grew on beaches fringing continental coastlines might easily be eliminated by grazing and browsing animals, overwhelmed by competing vegetation, desiccated by drought or destroyed by windstorm.

It is possible that these coconuts might have been found by people who migrated from Africa, via Asia to Australasia some 60 000 YBP. Not yet farmers they did not immediately take the palm into cultivation, but they could have used the immature fruit as a source of drinking water, accessible without using tools (Harries 1979) and the mature fruit as floatation aids when swimming across estuaries and bays, around headlands or in the open sea to offshore islands (Adkins *et al.* 2010).

### The ancient domestic habitat

A large area of land, mostly <1000 m above sea level, once connected the present-day Malaysian, Indonesian and Philippine islands to mainland southeast Asia and had a suitable climate for many food plants, including the coconut palm. This long-lived perennial (80 years or more) with its year round flowering and fruiting did not need frequent replanting. Slow and recalcitrant germination was no problem because the viviparous seednuts were simply allowed to grow where they fell, and they did not need to be dried for storage or for conversion to copra (which was not a traded item until the 19th century). It was only when ice sheets melted and sea levels rose during Mesolithic inter-glacial periods some 8 to 14 000 years ago that domestication is thought to have taken place in this southeast Asian region of Malesia (Harries 1990; Harries *et al.* 2004), which therefore represents a second centre of diversity. The process of selection was driven by extreme environmental changes, when the low-lying continental landmass of Sahul was submerged by rising sea levels, to become the Sulu and South China seas. Not only were coconuts better able to withstand tsunami-like flooding and the associated increasing soil salinity, but they also provided fresh, sweet, uncontaminated tender-nut water for thirsty human communities as wells and surface-water became too saline to drink.

At that time coconuts were not the crop that is known today. There was no demand for copra (the dried kernel) and other plants or animals could also provide fats and fibres for domestic households. But alone from any other plant, coconuts were a source of palatable, portable and potable (drinkable) water (Harries 1978). They became ‘the bottled-water on the supermarket shelf of life’.

#### Domestic selection

Unconscious human selection by the earliest cultivators would have included changes in plant habit, pest and disease resistance or windstorm tolerance, for example, but it was the tender-nut water content that was most important. For any given size or weight of fruit, there is a greater volume of water in the cavity of a spherical, rather than an ovoid nut. The kernel content increases in direct proportion to the increased water content and does so at the expense of reducing the proportion of the husk and shell components. The reduction in husk thickness at the ends of a long, angular fruit would result in a less angular, more spherical, fruit. Visual selection for this fruit shape is easy and would be applied automatically by adult or child, without conscious thought or effort, at every human and every coconut generation—and on every occasion when rising sea levels inundated domestic homesteads.

#### Germination rate

The resulting coconut fruit can still float, but rather too high in the water, and more importantly, the thinner husk allows the embryo to germinate more quickly taking from 30 to 140 days—and often beginning while still on the palm (Whitehead 1965). For instance, a study in the Solomons (Foale 1968) took 500 seednuts from each of three tall varieties (FMS, Rennell and Solomons) that were reaped when the husk had just begun to dry. This was considered to be the first sign of nut maturity, yet when the husks were carefully removed some 50 seednuts from each variety were found to have already germinated within the husk. Such early germination, which would be disastrous to long-distance oceanic dispersal by floating, has never been reported in the long, angular fruited type (Whitehead 1966) and there are no compelling arguments for any explanation involving domestic selection of a slow-germinating, long-fruited type from a quick-germinating round or intermediate form.

#### Locations

The emergence of the crow's beak and retention of the sprouted fruit high above ground level would make planting material available even when seednuts on the ground had been washed away by floods or seedlings destroyed by excessive salinity. This is surely the origin of the traditional techniques previously mentioned—to keep seednuts for germination on the roof of a thatched house, or tied in pairs with a strip of husk split from each and then suspended from branches of trees or bamboo posts, or tied round an upright pole—methods that are thus neither ‘odd’ nor ‘defective’. Indeed, under such circumstances, it is the ground nursery that would be unsatisfactory.

When making a Pacific germplasm collection, Whitehead (1966) recorded mature coconut fruit with ∼600–700 mL of water from some of the more isolated islands, specifically Rennell, Rotuma and Wallis (p. 48) and attributed their presence as a source of water for Polynesian travellers. The report also included a photograph (p. 70) of nuts tied around a pole in Western Samoa but the caption was ‘nuts for copra making’ rather than germination. This may have been a simple misunderstanding because, when copra became an industrial crop in the 19th and 20th centuries, ground nurseries generally became the preferred propagation method for commercial nurseries, first in south Asia and almost everywhere else, but providentially, not in Yap.

Drinking water has been essential to islanders and mariners from the very earliest times. The easily recognized large, spherical coconuts with high water content would have been carried in canoes as provisions for any journey, whether or not the people expected, in advance, to find coconut palms when they arrived at their destination. The pattern of distribution is clear—carried for food and drink, coconuts were taken not just to the isolated islands but to any island *en route* and the method to germinate them would have travelled simultaneously.

### The present-day cultivated habitats

Where populations of long, angular, thick-husked, slow-germinating coconuts might already have been present—on Indian Ocean islands like the Cocos-Keeling group (Leach *et al.* 2003) and on Pacific islands like Palmyra atoll (Rock 1916 cited by Sauer 1967)—their influence would predominate and an introduction of a few spherical, thin-husked, early germinating seednuts would not have been very significant. In contrast, even a small number of the domestic type could come to dominate the high islands like Rennell, Rotuma and Wallis mentioned previously, which would have had few, if any, naturally disseminated coconuts. However, when the two contrasting types did grow in one location they would introgress and populations with intermediate characteristics would be generated.

So, as the canoe voyages extended across the Pacific from island to island, the travellers might carry whatever was available; nevertheless it would be early germinators that would be put to one side in the canoe (probably tied to poles) to use as planting material at the next landfall. The notably unusual feature of the Yap germination technique—removing the haustorium and residual kernel—may even commemorate an unexpectedly prolonged passage, when their ancestors had been close to starvation. They would have been hungry enough to eat and drink what little they could get from the last remaining seednuts, knowing that the germinated seedlings represented their future source of food if they arrived on an uninhabited shore—a very real risk to Pacific mariners down the ages.

Whether or not such an event took place, it is certain that early travellers in the Pacific carried coconuts, and on islands like Tonga and Samoa it is possible to identify individual palms that have thick husk (*Niu kafa*) and others that have high water content (*Niu vai*) among general coconut populations with intermediate characteristics (so the Pacific islands are a third centre of diversity). However, recent DNA analyses show that coconuts did not reach the Pacific coast of America from Polynesian islands (Gunn *et al*. 2011). Not only is the distance is too great for coconuts to float and survive, there is no reliable evidence for the presence of Polynesian settlements, or any firm proof of pre-Columbian coconuts (Clement *et al.* 2013) and the genetic data identify the Philippines as the most likely source. A case can be made for a direct introduction from the Philippines to Mexico using the early germinating type in or soon after 1565 (Harries 2012) and for its spread from there to all points south to Peru.

It was the eventual entry of this type into the Caribbean after the opening of the Panama canal in 1915 (Harries 1971) that resulted in two distinct populations in Jamaica that allowed the significance of the speed of germination to be recognized (Whitehead 1965; Harries 1981). The Atlantic-Caribbean coconut populations are of the thick husk, slow germination category that can be sourced to East Africa or India. They could not float around the Cape of Good Hope but were carried to the Cape Verde islands by Portuguese explorers in 1499 and within 50 years to Puerto Rico and to Brazil and other locations soon after (Harries 1977). So it is the Atlantic-Caribbean (as a fourth centre of diversity) that is ‘the exception that tests the rule that slow germinators are dispersed by floating and quick germinators by boating’ and confirms the centres of diversity for slow germinators in the Indian Ocean, quick germinators in Malesia and introgressed types in the Pacific.

## Discussion

There may be other, more subtle, differences between the two fruited forms and their germination rates that concern their abscission when ripe because while the angular fruits naturally fall to the ground, the spherical fruits tend to remain on the bunches in the leaf axils of the palm. Quick germination is not the only selection criterion for the coconut palm. There were opportunities for cultivators and farmers to select palms with dwarf or compact habits, bright (red or yellow) fruit colours, aromatic or makapuno endosperm, and sweeter water or edible husk. There would also be unconscious selection for windstorm tolerance or pest and disease resistance, simply by planting survivors from hurricanes and epidemics. But once coconut became a commercial crop the nursery propagation methods became economically important and the various techniques to improve germination success, previously mentioned, were applied piecemeal. Now it may be possible to match different techniques to particular populations.

## Conclusions and forward look

At a time when *in vitro* embryo culture techniques are being adopted for the safe exchange and cryo-conservation of coconut germplasm, there are still many variations among standard commercial nursery germination techniques and traditional methods are not well known. Despite more than a century of agricultural cultivation preceded by some millennia of subsistence cultivation, germination speed and nursery techniques are different across the Indian Ocean and Pacific Ocean regions and it was in the Atlantic-Caribbean region that the differences first became apparent. These findings confirm recent DNA identification of two centres of coconut diversity, indicate how they might have arisen and suggest that the global distribution may now have four such centres. It will be necessary to test this claim with further DNA analysis and it is recommended that genetic markers related to husk thickness, fruit abscission and speed of germination should be implemented.

## Sources of funding

The work was funded by the Coconut Time Line (UK).

## Conflict of interest statement

None declared.

## References

[PLS045C56] Adkins S, Foale M, Harries H (2010). Growth and production of coconut. In: Verheye WH, ed. *Soils, plant
growth and crop production*. In: *Encyclopedia of Life Support Systems
(EOLSS)*, developed under the auspices of UNESCO. Oxford, UK: Eolss
Publishers. http://www.eolss.net.

[PLS045C1] Aiyadurai SG (1956). Observations on germination of dry seed coconuts. Madras Agricultural Journal.

[PLS045C2] **Anonymous** (1960). Yap's unique method of coconut cultivation. Micronesian Reporter.

[PLS045C3] Balachandran C, Arumughan C (1995). Biochemical and cytochemical transformations in germinating coconut
(*Cocos nucifera* Linn.). Journal of the American Oil Chemists’ Society.

[PLS045C4] Balasubramaniam K, Atukorala TMS, Wijesundera S, Hoover AA, da Silva MAT (1973). Biochemical changes during germination of the coconut (*Cocos
nucifera*). Annals of Botany.

[PLS045C5] Child R (1974). Coconuts.

[PLS045C6] Chin HF, Roberts EH (1980). Recalcitrant crop seeds.

[PLS045C7] Clement CR, Zizumbo-Villarreal D, Brown CH, Ward RG, Alves-Pereira A, Harries HC (2013). Coconuts in the Americas. Botanical Review.

[PLS045C8] Copeland EB (1914). The coconut.

[PLS045C9] Corner EJH (1966). The natural history of palms.

[PLS045C10] D'Amato A, Fasoli E, Righetti PG (2012). Harry Belafonte and the secret proteome of coconut milk. Journal of Proteomics.

[PLS045C11] de Guzman EV, del Rosario AG (1964). The growth and development of *Cocos nucifera* (Makapuno)
embryos *in vitro*. Philippine Agriculturist.

[PLS045C12] de Silva MAT, Aputharajah PP (1977). Micronutrients in the nutrition of coconut III. Effect of a supplementary
source of micronutrients on germination and growth of coconut seedlings. Ceylon Coconut Quarterly.

[PLS045C13] Foale MA (1968). Growth of the young coconut palm (*Cocos nucifera* L) 2. The
influence of nut size on seedling growth in three cultivars. Australian Journal of Agricultural Research.

[PLS045C14] Foale MA, Nair MK (1993). The effect of exposing the germpore on germination of
coconut. Advances in coconut research and development.

[PLS045C57] Gunn BF, Baudouin L, Olsen KM (2011). Independent origins of cultivated coconut (*Cocos nucifera*
L.) in the Old World Tropics. PLoS ONE.

[PLS045C15] Harries HC (1971). Coconut varieties in America. Oléagineux.

[PLS045C16] Harries HC (1977). The Cape Verde region (1499 to 1549); the key to coconut culture in the
Western Hemisphere. Turrialba.

[PLS045C17] Harries HC (1978). The evolution, dissemination and classification of *Cocos
nucifera*. Botanical Review.

[PLS045C18] Harries HC (1979). Nuts to the Garden of Eden. Principes (Journal of the International Palm Society).

[PLS045C19] Harries HC (1981). Germination and taxonomy of the coconut palm. Annals of Botany.

[PLS045C20] Harries HC (1983). A ten point coconut nursery programme to avoid germination
problems. Planter.

[PLS045C21] Harries HC, Baas P, Kalkman K, Geesink R (1990). Malesian origin for a domestic *Cocos
nucifera*. The plant diversity of Malesia.

[PLS045C22] Harries HC (2012). Key to coconut cultivation on the Pacific coast of America: the
Manila-Acapulco galleon route (1565–1815). Palms (Journal of the International Palm Society).

[PLS045C23] Harries H, Baudouin L, Cardeña R (2004). Floating, boating and introgression: molecular techniques and the ancestry
of coconut palm populations on Pacific Islands. Ethnobotany Research & Applications.

[PLS045C24] John CM, Narayana GV (1942). A simple method of preserving seed coconuts. Madras Agricultural Journal.

[PLS045C25] Kartha S (1981). Embryo of coconut and its germination. Journal of Plantation Crops.

[PLS045C26] Kenman ET (1973). Effect of seednut trimming on the germination and growth of
coconuts. Papua New Guinea Agricultural Journal.

[PLS045C27] Kirkwood JE, Gies WJ (1902). Chemical studies of the coconut with some notes on the changes during
germination. Bulletin of the Torrey Botanical Club.

[PLS045C28] Konan BR, Konan JL, Tetchi F, Assa RR, Amani G (2011). The biochemical characteristics of coconut (*Cocos nucifera*
L.) water during germination. International Journal of Biological and Chemical Sciences.

[PLS045C29] Leach B, Foale MA, Ashburner R (2003). Some characteristics of wild and managed coconut palm populations and their
environment in the Cocos (Keeling) Islands, Indian Ocean. Genetic Resources and Crop Evolution.

[PLS045C30] Manjula C, Chempakam B, Rajagopal V (1993). Solubilization and utilization of seed reserves during the germination of
coconut. Journal of Plantation Crops.

[PLS045C31] Manjula C, Chempakam B, Rajagopal V (1995a). Changes in nut water constitutents during seed germination in
coconut. Plant Physiology and Biochemistry.

[PLS045C32] Manjula C, Chempakam B, Rajagopal V (1995b). Germination, growth and dry matter partitioning in coconut. Philippine Journal of Coconut Studies.

[PLS045C33] Marar MMK, Jayarajan TG (1963). Coconut nursery studies: effect of the method of collecting seednuts on
germination of nuts and vigour of seedlings. Indian Coconut Journal.

[PLS045C34] Marar MMK, Shambhu K (1961). Coconut nursery studies III. Vigour of seedlings in relation to the
floating position of seednuts in water. Indian Coconut Journal.

[PLS045C35] Marar MMK, Varma R (1958). Coconut nursery studies. Effects of maturity of seednuts on germination and
vigour of seedlings. Indian Coconut Journal.

[PLS045C36] Menon KPV, Pandalai KM (1958). The coconut palm, a monograph.

[PLS045C37] N'Nan O, Borges M, Konan JL, Hocher V, Verdeil JL, Tregear J, N'guetta AS, Engelmann F, Malaurie B (2012). A simple protocol for cryopreservation of zygotic embryos of ten accessions
of coconut (*Cocos nucifera* L.). In Vitro Cellular and Developmental Biology—Plant.

[PLS045C38] Ohler JG (1999). Modern coconut management.

[PLS045C39] Pandittesekere G (1914). Coconut nurseries. Tropical Agriculturalist.

[PLS045C40] Pieris WVD (1937). Nursery management and selection of seedlings. Tropical Agriculturalist (Ceylon).

[PLS045C41] Pomier M. (1967).

[PLS045C42] Reddy DVS, Kumaran PM, Reddy LS (2001). Guidelines for establishing coconut seed garden and raising coconut
seedlings. Indian Coconut Journal.

[PLS045C43] Remison SU, Mgbeze CC (1988). Effects of storage and planting methods on the germination of
coconut. Nigerian Journal of Palms and Oil Seeds (Nigeria).

[PLS045C44] Rock JF (1916). Palmyra Island with a description of its flora. College of Hawaii Publications Bulletin.

[PLS045C45] Satyabalan K (1985). Nursery studies in West Coast Tall coconut. 3. Germination, seedling growth
and output of quality seedlings in relation to storage period of
seednuts. Indian Coconut Journal.

[PLS045C46] Sauer JD, Riley CL, Kelly JC, Pennington CW, Rands RL (1967). A re-evaluation of the coconut as an indicator of human
dispersal. Man across the sea.

[PLS045C47] Sugimura Y (1998). Ultrastructural observation of the haustorium in germinating
coconut. Japanese Journal of Tropical Agriculture.

[PLS045C48] Sumathykutty Amma B (1964). Preliminary studies on the effect of micronutrients on the germination of
coconut seednuts. Current Science.

[PLS045C49] Thomas KM (1974). Influence of certain physical and chemical treatments on the germination
and subsequent growth of coconut *Cocos nucifera* L seedlings: a
preliminary study. East African Agriculture and Forestry Journal.

[PLS045C50] Ugbah MM, Akpan EEJ (2003). Effect of nut positioning and husk slashing of nut germination and growth
of two coconut varieties. Nigerian Journal of Palms and Oil Seeds.

[PLS045C51] Whitehead RA (1965). Speed of germination, a characteristic of possible taxonomic significance
in *Cocos nucifera* L. Tropical Agriculture (Trinidad).

[PLS045C52] Whitehead RA (1966). Sample survey and collection of coconut germplasm in the Pacific islands (30
May–5 September 1964).

[PLS045C53] Whitehead RA (1968). Collection of coconut germplasm from the Indian/Malaysian region, Peru and the
Seychelles islands and testing for resistance to lethal yellowing disease.

[PLS045C54] Winton AL (1901). The anatomy of the fruit of *Cocos nucifera*. American Journal of Science.

[PLS045C55] Zuniga LC, Armedilla A, Alimagno L, Ala DS, de Gala D, Penaflorida G, Sahagun A (1971). A note on the effects of coconut water transfusion on the germination and
growth of waterless coconuts. Philippine Journal of Plant Industry.

